# European Public Health News

**DOI:** 10.1093/eurpub/ckac187.001

**Published:** 2023-02-03

**Authors:** Marieke Verschuuren, Satish Mishra, Hedinn Halldorsson, Natasha Azzopardi Muscat, Faith Kilford Vorting, Hans Henri P Kluge, Anthony Staines, Floris Barnhoorn

**Affiliations:** (EUPHA); (EUPHA); (EUPHA); (EUPHA); (EUPHA); (EUPHA); (EPH Conference); (EPH Conference)

In this first 2023 edition of the European Public Health News, the EUPHA office column focuses on EUPHA’s governance structures. Although seen by many as a ‘necessary evil’, the governance structures are at the heart of the EUPHA organization and thus very important for EUPHA. Mishra *et al*. of the WHO Regional Office for Europe put the spotlight on rehabilitation, a health systems function that unfairly often is seen as non-essential. As a consequence, millions of people in Europe do not have access to necessary rehabilitation care. Finally, Staines and Barnhoorn look ahead at the European Public Health (EPH) Conference in Dublin in November this year. The theme of this 16th EPH Conference is Our Food, Our Health, Our Earth: A Sustainable Future for Humanity.

## EUPHA office column: the importance of our governance structures

VerschuurenMarieke(EUPHA)

Governance structures and procedures may not be the most sexy topic in the eyes of many, nevertheless, I have chosen to dedicate this edition of the EUPHA office column to it. Why? Because EUPHA’s governance bodies are essential to EUPHA. They determine EUPHA’s functioning, relevance and impact. Basically, to a large extent, the governing bodies ‘are’ EUPHA.

### EUPHA Governing Board and Executive Council

At the heart of our organization is the Governing Board. The Governing Board consists of representatives of our members—national public health associations and institutions—and thereby is EUPHA’s highest body. It provides guidance for EUPHA’s strategic direction and approves the EUPHA’s 5-year strategies and work plans, the annual financial and programmatic reports, and the work plan and budget for the coming year. Last but not least, the Governing Board appoints the members of the Executive Council. The Executive Council is charged with the management of the association.

### Welcome to our new EUPHA President-elect and Secretary of the Executive Council

EUPHA works with a ‘three presidents-system’: next to the current president, the immediate past president and the president-elect are also part of the Executive Council. This ensures continuity and smooth transitions between presidencies. During EUPHA’s Governing Board meeting in November last in Berlin, the Governing Board has elected the new EUPHA President-elect: Dr. Tit Albreht. Dr. Albreht works at the National Institute of Public Health of Slovenia and is an expert in health services research and cancer control. The Governing Board also elected a new Secretary of the Executive Council in November: Prof. Charles Agyemang. Prof. Agyemang is a professor of Global Migration, Ethnicity and Health at Amsterdam University Medical Centres. Both Tit and Charles have long track-records in European public health and know EUPHA well, and we are therefore very pleased that they are now part of our Executive Council. Welcome Tit and Charles!

### Section Council

EUPHA sections bring together researchers, policymakers and practitioners working in the same field for knowledge sharing and capacity building. The sections are a vital element of the EUPHA organization, as they bring forward the evidence-base that EUPHA disseminates and uses in its advocacy work. There currently are 26 operational sections. Daily operations of the sections are in the hands of the section presidents, vice-presidents and steering committees. The section presidents form the Section Council. The Section Council represents the interests and common concerns of all sections, facilitates relations between the sections and other relevant bodies of the association, and makes recommendations to the annual conference program.

While I am writing this column, 2022 is drawing to a close and 2023 is about to kick off. We are extremely grateful to all our member associations and institutions, the Governing Board, the Executive Council, and the Section Council and other active section members for all their commitment to EUPHA. Without their contributions, EUPHA would not be where it is right now. We look forward to a new year filled with impactful collaborations, scientific advancement and international friendship!

## Rehabilitation: The ignored, growing need of 400 million people

MishraSatish(EUPHA)HalldorssonHedinn(EUPHA)MuscatNatasha Azzopardi(EUPHA) Kilford VortingFaith(EUPHA)KlugeHans Henri P(EUPHA)

Of the almost 1 billion people who live in the WHO European Region, 400 million were in need of rehabilitation in 2019. That is two in five people, the equivalent of the entire population of eight countries: Türkiye, Germany, France, the UK, Kazakhstan, Romania, Poland and the Netherlands. It represents 49 million years of healthy life lost.

Across the Region, some of the most common conditions that require rehabilitation include low back pain, fractures, hearing and vision loss, as well as stroke and dementia. Rehabilitation services support someone with a health condition to improve and maintain their ability to function, addressing impairments such as pain or muscle weakness and limitations in activities, such as self-care or walking.

The fact that 394 million people have a health condition amenable to rehabilitation, is staggering. Even more shocking is the fact that the majority of those requiring these services are not getting them.

Health equity varies in the 53 countries of our Region. There is a huge difference in access to and quality of rehabilitation services from one country to another. For example, there are 12 times fewer physiotherapists, 141 times fewer occupational therapists and 6 times fewer prosthetics and orthotics professionals in middle-income countries compared to higher-income ones in the Region. Yet for all countries, there is a dire need for rehabilitation services. And the main drivers of this rapidly growing need are an ageing population and the rise of non-communicable diseases.

These are the findings of a new WHO report. What is concerning, is that the report’s estimates were calculated based on 2019 data, before the COVID-19 pandemic and the war in Ukraine. It also acknowledges that rehabilitation is not seen an essential component of a medical response to health emergencies.

In May 2020, early on in the pandemic, 79% of surveyed countries reported disruptions in their rehabilitation services. There were several reasons for disrupted or terminated services, one of these being that rehabilitation was and is considered non-essential. Three years on, the debilitating symptoms of Long COVID and its impact on people’s functional ability, as well as the unfolding humanitarian crisis in Ukraine, are compounding rehabilitation needs in the Region.

These trends should urge health policy planners to prioritize rehabilitation services and integrate them into primary health. In far too many countries, rehabilitation is not fully or effectively integrated into primary health care or it is provided in secondary and tertiary level facilities in urban areas, leaving rural, remote areas underserved. This needs to change.

Rehabilitation helps people and communities to thrive. Most of us will require it at some point in our lives or know someone who already does. So why aren’t we realizing its potential? And how do we change our priorities?

A key barrier is the lack of knowledge or awareness about rehabilitation, its benefits, and how it works. Misconceptions regarding affordability are another barrier. For this reason, WHO urges countries to prioritize rehabilitation and define it as an essential service; integrate it at all levels of healthcare systems, and develop an adequate, well-trained workforce to deliver rehabilitation services.

But first of all, we need to change how we think about rehabilitation. It is not a non-priority, it is not a disability-specific service, and it is not a luxury nor an optional service. In fact, it supports economic prosperity. It helps deliver and maintain the best outcomes of other health interventions. It has the power to unleash significant human potential, contribute to societal growth and improve the well-being of many millions. It has a life-transforming impact on individuals, reduces pain, supports mental well-being, empowers people and enables participation in work, education and society.

Rehabilitation has been down-prioritized and under-resourced for far too long. It is time to change that. The sheer size of the need should dispel the misconception that rehabilitation is a service required by only a select few, unaffordable to most. Rehabilitation is a crucial, cost-effective element of achieving universal health coverage, and we can all contribute to making this happen.

## Our Food, Our Health, Our Earth: A Sustainable Future for Humanity

StainesAnthony
(EPH Conference)
BarnhoornFloris
(EPH Conference)

16th European Public Health Conference

8–11 November 2023

Convention Centre Dublin (The CCD), Dublin, Ireland

### Welcome to Dublin 2023

After the successful conference in Berlin, we will meet again in person for the 16th EPH conference in Dublin from 9 to 12 November. The spectacular Convention Centre Dublin (The CCD) provides us with ample opportunities for informal chatting, personal exchange and live networking. Recognized as the first carbon neutral constructed convention centre in the world, The CCD’s contemporary design meets the highest standards of environmental sustainability. We hope you will join us and we look forward to welcoming you in Dublin.

### Abstract submission for Dublin 2023

EPH Conferences offer excellent opportunities to learn from the latest research and practice, to network with experts and colleagues and to expand your professional horizons. Why don’t you participate at Dublin 2023 by sharing your work with colleagues? Abstracts are invited for workshops and single presentations in all areas of public health research, practice, policy and education. Abstract submission is open from 1 February to 1 May 2023. For less experienced abstract submitters a Tutoring Programme is set-up. Detailed instructions can be found on www.ephconference.eu.

### Our Food, Our Health, Our Earth: A Sustainable Future for Humanity

The theme for Dublin 2023 is ‘Our Food, Our Health, Our Earth: A Sustainable Future for Humanity’. Over the next decade, we will all make choices which will determine the future of our advanced technological society. The COVID-19 and mpox pandemics are as examples of the instability of our way of life, and the need for action on One Health. In Europe, we have paid a high price for our belief that we were safe from infection, which we could dismantle our public health systems, and get away with it. Other countries to whom COVID-19 spread from Europe have suffered more, and paid even higher prices. We now have no way out in terms of climate emergency. The global climate is rapidly deteriorating. COP27 has taken an important step towards bringing justice to those on the front lines who have done so little to cause the climate crisis. But little was achieved in the ambition to keep the 1.5 degree limit alive and pull humanity back from the climate cliff.

The Dublin 2023 conference hopes to open up part of this discussion, with a focus on health, and health care. We will look specifically at global health, the use of digital tools, equity in health, and the workforce skills to provide good care. We will, in line with the overall conference theme, look in depth at the climate emergency, and very specifically at the human food supply. As we have seen recently, Russia’s invasion of Ukraine poses serious risks to global food security. The unfolding crisis in Ukraine will push up already-high food price inflation. This will have serious consequences for low-income net-food importing countries, many of which have seen already an increase in malnourishment rates in the face of the pandemic. The Ukraine conflict not only exposes the ruthlessness of the current Russian government but also our failure to create a resilient sustainable global food supply. We can do better, and we have to do better.

We look forward to seeing you all in person, in Dublin.

Anthony Staines, Chair 16th EPH Conference 2023

Floris Barnhoorn, EPH Conference Office

